# 6-(4-Meth­oxy­phen­yl)-1,3,5-triazine-2,4-diamine

**DOI:** 10.1107/S1600536812038019

**Published:** 2012-09-08

**Authors:** Kaliyaperumal Thanigaimani, Ibrahim Abdul Razak, Suhana Arshad, Rathinavel Jagatheesan, K. Joseph Santhanaraj

**Affiliations:** a School of Physics, Universiti Sains Malaysia, 11800 USM, Penang, Malaysia; b Department of Chemistry, Angalamman College of Engineering and Technology, Siruganur, Tiruchirappalli 621 105, Tamil Nadu, India; c Department of Chemistry, St. Joseph’s College, Tiruchirappalli 620 002, Tamil Nadu, India

## Abstract

In the title compound, C_10_H_11_N_5_O, the triazine ring forms a dihedral angle of 10.37 (4)° with the benzene ring. In the crystal, adjacent mol­ecules are linked by a pair of N—H⋯N hydrogen bonds, forming an inversion dimer with an *R*_2_^2^(8) ring motif. The dimers are further connected *via* N—H⋯O and N—H⋯N hydrogen bonds, resulting in a three-dimensional network.

## Related literature

For the biological activity of triazine derivatives, see: Bork *et al.* (2003 ▶). For hydrogen-bond motifs, see: Bernstein *et al.* (1995 ▶). For the stability of the temperature controller used in the data collection, see: Cosier & Glazer (1986 ▶).
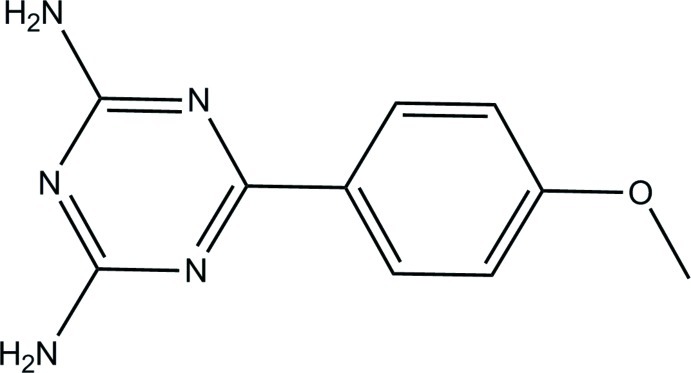


## Experimental

### 

#### Crystal data

C_10_H_11_N_5_O
*M* *_r_* = 217.24
Monoclinic, 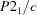

*a* = 7.4340 (2) Å
*b* = 10.0355 (3) Å
*c* = 14.6803 (4) Å
β = 114.191 (1)°
*V* = 999.03 (5) Å^3^
*Z* = 4
Mo *K*α radiation
μ = 0.10 mm^−1^
*T* = 100 K
0.73 × 0.49 × 0.15 mm

#### Data collection

Bruker SMART APEXII CCD area-detector diffractometer
Absorption correction: multi-scan (*SADABS*; Bruker, 2009 ▶) *T*_min_ = 0.930, *T*_max_ = 0.985
16310 measured reflections
3611 independent reflections
3112 reflections with *I* > 2σ(*I*)
*R*_int_ = 0.024

#### Refinement

*R*[*F* ^2^ > 2σ(*F* ^2^)] = 0.041
*wR*(*F* ^2^) = 0.121
*S* = 1.07
3611 reflections
162 parameters
H atoms treated by a mixture of independent and constrained refinement
Δρ_max_ = 0.50 e Å^−3^
Δρ_min_ = −0.26 e Å^−3^

### 

Data collection: *APEX2* (Bruker, 2009 ▶); cell refinement: *SAINT* (Bruker, 2009 ▶); data reduction: *SAINT*; program(s) used to solve structure: *SHELXTL* (Sheldrick, 2008 ▶); program(s) used to refine structure: *SHELXTL*; molecular graphics: *SHELXTL*; software used to prepare material for publication: *SHELXTL* and *PLATON* (Spek, 2009 ▶).

## Supplementary Material

Crystal structure: contains datablock(s) global, I. DOI: 10.1107/S1600536812038019/is5191sup1.cif

Structure factors: contains datablock(s) I. DOI: 10.1107/S1600536812038019/is5191Isup2.hkl

Supplementary material file. DOI: 10.1107/S1600536812038019/is5191Isup3.cml

Additional supplementary materials:  crystallographic information; 3D view; checkCIF report

## Figures and Tables

**Table 1:** Hydrogen-bond geometry (Å, °)

*D*—H⋯*A*	*D*—H	H⋯*A*	*D*⋯*A*	*D*—H⋯*A*
N2—H2*B*⋯N5^i^	0.878 (14)	2.258 (14)	3.1291 (11)	172.1 (12)
N4—H4*A*⋯N3^ii^	0.894 (16)	2.077 (16)	2.9708 (12)	177.4 (14)
N4—H4*B*⋯O1^iii^	0.879 (16)	2.189 (15)	3.0196 (11)	157.3 (14)

Symmetry codes: (i) 
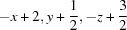
; (ii) 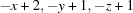; (iii) 
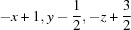
.

**Fig. 1.**
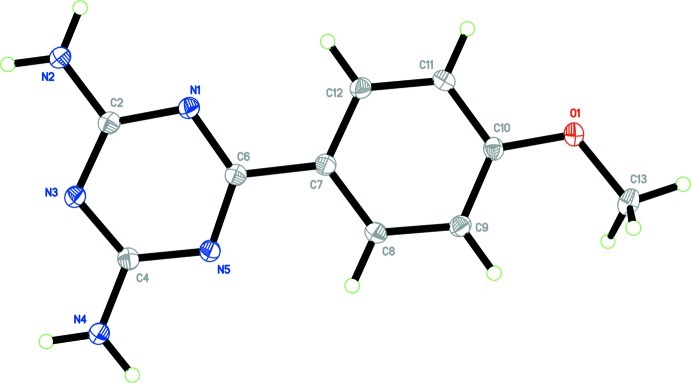
The molecular structure of the title compound, showing 50% probability displacement ellipsoids and the atom-numbering scheme.

**Fig. 2.**
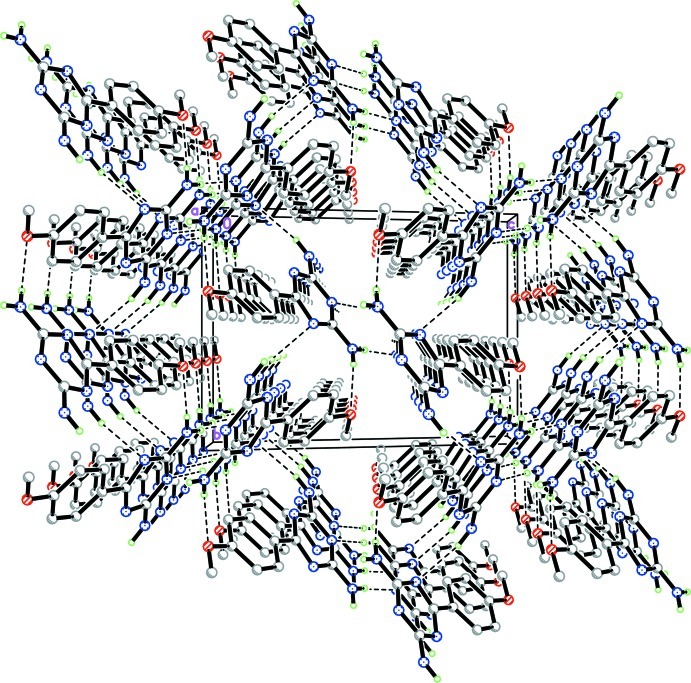
The crystal packing of the title compound. The H atoms not involved in the intermolecular interactions (dashed lines) have been omitted for clarity.

## supplementary crystallographic information

### Comment

Triazine derivatives show antitumour activity, as well as a broad range of biological activities, such as anti-angiogenesis and antimicrobial effects (Bork *et al.*, 2003). Herein, we report the crystal structure determination of the title compound, (I).

The asymmetric unit of the title compound is shown in Fig. 1. The essentially planar triazine ring [N1/C2/N3/C4/N5/C6, maximum deviation of 0.036 (1) Å at atom C2] forms a dihedral angle of 10.39 (4)° with the benzene ring (C7–C12). In the crystal structure, molecules are linked by a pair of N4—H4A···N3^ii^ hydrogen bonds (symmetry code in Table 1), forming an *R*_2_^2^(8) (Bernstein *et al.*, 1995) ring motif and an inversion dimer (Fig. 2). The dimers are further connected *via* N4—H4B···O1^iii^ and N2—H2B···N5^i^ hydrogen bonds (symmetry codes in Table 1), resulting into a three-dimensional network.

### Experimental

Hot methanol solution (20 ml) of 2,4-diamino-6-(4-methoxyphenyl)-1,3,5-triazine (32 mg Aldrich) was warmed for a half an hour over a water bath. The resulting solution was allowed to cool slowly at room temperature. After a few days colourless plate-like crystals were obtained.

### Refinement

N-bound H atoms were located in a difference Fourier maps and refined freely [refined N—H distances 0.896 (15), 0.877 (14), 0.896 (15) and 0.878 (15) Å]. The remaining H atoms were positioned geometrically (C—H = 0.95–0.98 Å) and were refined using a riding model, with *U*_iso_(H)=1.2*U*_eq_(C) and 1.5*U*_eq_(methyl C). A rotating-group model was used for the methyl group.

### Crystal data

**Table:**

C_10_H_11_N_5_O	*F*(000) = 456
*M**_r_* = 217.24	*D*_x_ = 1.444 Mg m^−^^3^
Monoclinic, *P*2_1_/*c*	Mo *K*α radiation, λ = 0.71073 Å
Hall symbol: -P 2ybc	Cell parameters from 7910 reflections
*a* = 7.4340 (2) Å	θ = 2.5–32.5°
*b* = 10.0355 (3) Å	µ = 0.10 mm^−^^1^
*c* = 14.6803 (4) Å	*T* = 100 K
β = 114.191 (1)°	Plate, colourless
*V* = 999.03 (5) Å^3^	0.73 × 0.49 × 0.15 mm
*Z* = 4	

### Data collection

**Table:**

Bruker SMART APEXII CCD area-detector diffractometer	3611 independent reflections
Radiation source: fine-focus sealed tube	3112 reflections with *I* > 2σ(*I*)
Graphite monochromator	*R*_int_ = 0.024
φ and ω scans	θ_max_ = 32.6°, θ_min_ = 2.5°
Absorption correction: multi-scan (*SADABS*; Bruker, 2009)	*h* = −11→11
*T*_min_ = 0.930, *T*_max_ = 0.985	*k* = −15→14
16310 measured reflections	*l* = −22→22

### Refinement

**Table:**

Refinement on *F*^2^	Primary atom site location: structure-invariant direct methods
Least-squares matrix: full	Secondary atom site location: difference Fourier map
*R*[*F*^2^ > 2σ(*F*^2^)] = 0.041	Hydrogen site location: inferred from neighbouring sites
*wR*(*F*^2^) = 0.121	H atoms treated by a mixture of independent and constrained refinement
*S* = 1.07	*w* = 1/[σ^2^(*F*_o_^2^) + (0.0686*P*)^2^ + 0.2052*P*] where *P* = (*F*_o_^2^ + 2*F*_c_^2^)/3
3611 reflections	(Δ/σ)_max_ < 0.001
162 parameters	Δρ_max_ = 0.50 e Å^−^^3^
0 restraints	Δρ_min_ = −0.26 e Å^−^^3^

### Special details

**Table:**

Experimental. The crystal was placed in the cold stream of an Oxford Cryosystems Cobra open-flow nitrogen cryostat (Cosier & Glazer, 1986) operating at 100.0 (1) K.
Geometry. All e.s.d.'s (except the e.s.d. in the dihedral angle between two l.s. planes) are estimated using the full covariance matrix. The cell e.s.d.'s are taken into account individually in the estimation of e.s.d.'s in distances, angles and torsion angles; correlations between e.s.d.'s in cell parameters are only used when they are defined by crystal symmetry. An approximate (isotropic) treatment of cell e.s.d.'s is used for estimating e.s.d.'s involving l.s. planes.
Refinement. Refinement of *F*^2^ against ALL reflections. The weighted *R*-factor *wR* and goodness of fit *S* are based on *F*^2^, conventional *R*-factors *R* are based on *F*, with *F* set to zero for negative *F*^2^. The threshold expression of *F*^2^ > σ(*F*^2^) is used only for calculating *R*-factors(gt) *etc*. and is not relevant to the choice of reflections for refinement. *R*-factors based on *F*^2^ are statistically about twice as large as those based on *F*, and *R*- factors based on ALL data will be even larger.

### Fractional atomic coordinates and isotropic or equivalent isotropic displacement parameters (Å^2^)

**Table:**

	*x*	*y*	*z*	*U*_iso_*/*U*_eq_	
O1	0.35597 (9)	0.64750 (7)	0.95775 (5)	0.01650 (14)	
N1	0.89236 (10)	0.73352 (7)	0.71745 (5)	0.01370 (14)	
N2	1.14046 (11)	0.80754 (8)	0.67477 (6)	0.01588 (15)	
N3	0.98420 (10)	0.61218 (7)	0.60228 (5)	0.01364 (14)	
N4	0.81934 (12)	0.41721 (8)	0.53671 (6)	0.01771 (16)	
N5	0.74625 (11)	0.52210 (7)	0.65619 (5)	0.01434 (15)	
C2	1.00276 (12)	0.71420 (8)	0.66483 (6)	0.01287 (15)	
C4	0.85102 (12)	0.51935 (8)	0.59951 (6)	0.01342 (15)	
C6	0.76980 (11)	0.63268 (8)	0.71082 (6)	0.01251 (15)	
C7	0.65370 (12)	0.63948 (8)	0.77219 (6)	0.01286 (15)	
C8	0.50541 (12)	0.54629 (9)	0.75799 (6)	0.01468 (16)	
H8A	0.4749	0.4824	0.7061	0.018*	
C9	0.40092 (12)	0.54448 (8)	0.81785 (6)	0.01421 (16)	
H9A	0.3015	0.4796	0.8078	0.017*	
C10	0.44520 (12)	0.63999 (8)	0.89293 (6)	0.01308 (15)	
C11	0.59087 (12)	0.73581 (9)	0.90702 (6)	0.01452 (16)	
H11A	0.6182	0.8016	0.9575	0.017*	
C12	0.69527 (12)	0.73512 (8)	0.84777 (6)	0.01394 (16)	
H12A	0.7954	0.7996	0.8583	0.017*	
C13	0.19727 (13)	0.55633 (10)	0.94259 (7)	0.01802 (17)	
H13A	0.1488	0.5697	0.9947	0.027*	
H13B	0.2448	0.4646	0.9457	0.027*	
H13C	0.0900	0.5724	0.8770	0.027*	
H2A	1.236 (2)	0.7853 (15)	0.6551 (11)	0.031 (4)*	
H2B	1.1747 (19)	0.8611 (14)	0.7264 (10)	0.023 (3)*	
H4A	0.882 (2)	0.4077 (15)	0.4965 (11)	0.029 (3)*	
H4B	0.739 (2)	0.3525 (15)	0.5360 (11)	0.030 (4)*	

### Atomic displacement parameters (Å^2^)

**Table:**

	*U*^11^	*U*^22^	*U*^33^	*U*^12^	*U*^13^	*U*^23^
O1	0.0177 (3)	0.0169 (3)	0.0189 (3)	−0.0022 (2)	0.0116 (2)	−0.0023 (2)
N1	0.0149 (3)	0.0116 (3)	0.0165 (3)	−0.0003 (2)	0.0083 (2)	−0.0002 (2)
N2	0.0171 (3)	0.0136 (3)	0.0197 (3)	−0.0030 (3)	0.0103 (3)	−0.0022 (3)
N3	0.0151 (3)	0.0123 (3)	0.0149 (3)	−0.0006 (2)	0.0075 (2)	0.0002 (2)
N4	0.0216 (3)	0.0152 (3)	0.0216 (3)	−0.0053 (3)	0.0143 (3)	−0.0051 (3)
N5	0.0163 (3)	0.0121 (3)	0.0174 (3)	−0.0008 (3)	0.0098 (3)	−0.0011 (2)
C2	0.0131 (3)	0.0114 (3)	0.0139 (3)	0.0015 (3)	0.0054 (3)	0.0021 (3)
C4	0.0146 (3)	0.0120 (3)	0.0143 (3)	0.0013 (3)	0.0064 (3)	0.0008 (3)
C6	0.0128 (3)	0.0111 (3)	0.0137 (3)	0.0014 (3)	0.0055 (3)	0.0013 (3)
C7	0.0134 (3)	0.0108 (3)	0.0154 (3)	0.0011 (3)	0.0070 (3)	0.0002 (3)
C8	0.0156 (3)	0.0128 (3)	0.0170 (3)	−0.0004 (3)	0.0081 (3)	−0.0025 (3)
C9	0.0137 (3)	0.0122 (3)	0.0177 (3)	−0.0007 (3)	0.0074 (3)	−0.0007 (3)
C10	0.0130 (3)	0.0128 (3)	0.0145 (3)	0.0020 (3)	0.0066 (3)	0.0012 (3)
C11	0.0159 (3)	0.0133 (4)	0.0154 (3)	−0.0009 (3)	0.0074 (3)	−0.0025 (3)
C12	0.0139 (3)	0.0119 (3)	0.0168 (3)	−0.0003 (3)	0.0069 (3)	−0.0004 (3)
C13	0.0157 (3)	0.0206 (4)	0.0199 (4)	−0.0016 (3)	0.0095 (3)	0.0022 (3)

### Geometric parameters (Å, º)

**Table:**

O1—C10	1.3669 (10)	C6—C7	1.4826 (11)
O1—C13	1.4364 (11)	C7—C8	1.3946 (12)
N1—C6	1.3384 (11)	C7—C12	1.4025 (12)
N1—C2	1.3517 (10)	C8—C9	1.3911 (11)
N2—C2	1.3507 (11)	C8—H8A	0.9500
N2—H2A	0.896 (15)	C9—C10	1.3945 (12)
N2—H2B	0.877 (14)	C9—H9A	0.9500
N3—C2	1.3441 (11)	C10—C11	1.3988 (12)
N3—C4	1.3479 (11)	C11—C12	1.3828 (11)
N4—C4	1.3335 (11)	C11—H11A	0.9500
N4—H4A	0.896 (15)	C12—H12A	0.9500
N4—H4B	0.878 (15)	C13—H13A	0.9800
N5—C6	1.3380 (11)	C13—H13B	0.9800
N5—C4	1.3530 (10)	C13—H13C	0.9800
			
C10—O1—C13	117.33 (7)	C9—C8—C7	121.75 (8)
C6—N1—C2	113.87 (7)	C9—C8—H8A	119.1
C2—N2—H2A	117.2 (10)	C7—C8—H8A	119.1
C2—N2—H2B	117.2 (9)	C8—C9—C10	118.57 (8)
H2A—N2—H2B	116.3 (13)	C8—C9—H9A	120.7
C2—N3—C4	114.56 (7)	C10—C9—H9A	120.7
C4—N4—H4A	123.0 (10)	O1—C10—C9	124.29 (7)
C4—N4—H4B	120.3 (9)	O1—C10—C11	115.23 (7)
H4A—N4—H4B	116.6 (13)	C9—C10—C11	120.47 (7)
C6—N5—C4	114.75 (7)	C12—C11—C10	120.24 (8)
N3—C2—N2	117.59 (7)	C12—C11—H11A	119.9
N3—C2—N1	125.68 (7)	C10—C11—H11A	119.9
N2—C2—N1	116.72 (7)	C11—C12—C7	120.17 (8)
N4—C4—N3	118.14 (7)	C11—C12—H12A	119.9
N4—C4—N5	117.24 (7)	C7—C12—H12A	119.9
N3—C4—N5	124.62 (8)	O1—C13—H13A	109.5
N5—C6—N1	126.06 (7)	O1—C13—H13B	109.5
N5—C6—C7	115.89 (7)	H13A—C13—H13B	109.5
N1—C6—C7	118.00 (7)	O1—C13—H13C	109.5
C8—C7—C12	118.78 (7)	H13A—C13—H13C	109.5
C8—C7—C6	119.98 (7)	H13B—C13—H13C	109.5
C12—C7—C6	121.18 (7)		

### Hydrogen-bond geometry (Å, º)

**Table:**

*D*—H···*A*	*D*—H	H···*A*	*D*···*A*	*D*—H···*A*
N2—H2*B*···N5^i^	0.878 (14)	2.258 (14)	3.1291 (11)	172.1 (12)
N4—H4*A*···N3^ii^	0.894 (16)	2.077 (16)	2.9708 (12)	177.4 (14)
N4—H4*B*···O1^iii^	0.879 (16)	2.189 (15)	3.0196 (11)	157.3 (14)

Symmetry codes: (i) −*x*+2, *y*+1/2, −*z*+3/2; (ii) −*x*+2, −*y*+1, −*z*+1; (iii) −*x*+1, *y*−1/2, −*z*+3/2.

## References

[bb1] Bernstein, J., Davis, R. E., Shimoni, L. & Chang, N.-L. (1995). *Angew. Chem. Int. Ed. Engl.* **34**, 1555–1573.

[bb2] Bork, J. T., Lee, J. W., Khersonsky, S. M., Moon, H. S. & Chang, Y. T. (2003). *Org. Lett.* **5**, 117–120. 10.1021/ol027195v 12529119

[bb3] Bruker (2009). *SADABS*, *APEX2* and *SAINT* Bruker AXS Inc., Madison, Wisconsin, USA.

[bb4] Cosier, J. & Glazer, A. M. (1986). *J. Appl. Cryst.* **19**, 105–107.

[bb5] Sheldrick, G. M. (2008). *Acta Cryst.* A**64**, 112–122. 10.1107/S0108767307043930 18156677

[bb6] Spek, A. L. (2009). *Acta Cryst.* D**65**, 148–155. 10.1107/S090744490804362X PMC2631630 19171970

